# What does it mean to be a physician? Exploring social imaginaries of first-year medical students

**DOI:** 10.5116/ijme.5e30.8f73

**Published:** 2020-03-27

**Authors:** Rachel Vaizer, Sanah Aslam, William G. Pearson Jr, Nicole Rockich-Winston

**Affiliations:** 1 Medical College of Georgia, Augusta University, USA; 2 The Human Flourishing Program, Institute for Quantitative Social Science, Harvard University, USA; 3 Department of Pharmacology and Toxicology, Medical College of Georgia, Augusta University, USA

**Keywords:** Social imaginary, social contract, medically underserved community, professional identity, patient-physician relationship

## Abstract

**Objectives:**

To explore if community embedded discussions with local community members reshape the social imaginary of medicine among students and contribute positively to their professional identity

**Methods:**

This explorative, qualitative study involved 35 first-year medical students who volunteered to attend a 2-hour forum at a local church to ask community members about their experiences with doctors and healthcare systems.  Student participants were asked to reflect on five structured questions. The written reflections were submitted for analysis, de-identified, and analyzed using Glaser’s classic grounded theory, constant comparative analysis, and Taylor’s model of modern social imaginaries as an analytical lens.

**Results:**

The results indicate that student participants identified seven main themes regarding what community members expect from their doctors, including active listening (n=22), physical touch (n=18), and compassion (n=16). Responses also indicated that only 5.6% of the students felt that the preclinical curriculum was adequately preparing them for what local community members identified as important to patient care. However, students recognized that two aspects of the curriculum, Physical Diagnosis (n=12) and volunteering/community engagement (n=9), were congruent with the expectations of future patients.

**Conclusions:**

The results suggest that students identified educational experiences that were congruent with the social imaginary of patients. However, patient expectations were discordant to some aspects of the medical imaginary of medical students. The experience and subsequent reflections may be salient to contributing to each student’s professional identity and provide a model for other medical schools to explore how the curriculum is fulfilling the community’s perception of ideal patient care.

## Introduction

The patient-physician relationship is foundational to the social contract between medicine and society.^1^ A physician’s commitment to the public good by putting each patient first is the basis of medical professionalism.^2^ While this long-withstanding arrangement has been criticized for perpetuating privilege,^3^ the disconnect between the healthcare systems and community members may also be attributed to a mutual misconception of expectations and aims between doctors and their patients.^4^ Rather than cancel medicine’s social contract as Harris proposes,^4^ involving the local community early in the training of medical students may provide substantial opportunities to solidify these obligations through enhancing the social imaginary of the trainee.

Although theorized and developed outside the field of medicine in the 1980s,^5^ Charles Taylor proposed the concept of a modern social imaginary as “the way we imagine our society.”^6^ The term imaginary is used to describe the values and practices that are important to a social group within societies and how it unites this group with a common purpose.^6^^,^^7^ This society as a whole interacts with, contributes to, and benefits from the imaginary with its associated social practices.^6^^,^^7^  Each imaginary carries with it a sense of how interactions generally proceed and an idea of how they should go that further legitimizes the community and its practices.^7^  Focusing attention on the perceived social imaginary of students then is critical for the development of their professional identity as well as for the common good.

In today’s healthcare system, the healthcare team is a conglomerate of many integral components – the patients, the physicians, the nurses, the pharmacists, the students, etc. Each element of the team has distinctive values and goals that unite them and build their social imaginary, underneath an encompassing imaginary of the healthcare in general. For example, the social imaginary of the patients accessing care likely includes expectations for a relationship with a clinician and outcomes of their care, while the social imaginary of the healthcare team may center on how to manage the patient’s health efficiently. These social imaginaries merge to create a lived experience of medicine as a whole.^6^^,^^7^

Literature exploring the social imaginaries of medical students and physicians has been limited to the intersectionality of personal spirituality and professional development.^8^^,^^9^ Importantly, however, previous studies exploring this intersectionality demonstrate that medical students can develop an alternate social imaginary that coincides with modern medicine. To our knowledge, exploring how medical students develop a patient-centered professional identity despite the disconnect between healthcare systems and community members has not been investigated. To address the discrepancy, this study is the first qualitative study to investigate a cohort of students who listened to patients in a community-embedded forum discuss their positive and negative experiences with physicians and the healthcare system. This study sought to expand on the previous research regarding intersectionality and broaden to include the perceptions of patients. This study aimed to explore whether discussions with local community members reshape the social imaginary of medicine among students and contribute positively to their professional identity.

## Methods

### Study Design

In this study, we employed an exploratory qualitative research design. This research method is commonly used to investigate issues that have not been studied or well-defined in the literature.^10^  The intervention involved medical students participating in a 2-hour open forum allowing community members at a local church to describe their interactions with the healthcare system, what types of characteristics they look for in their physicians, and how they feel the medical community can better serve them.

### Study participants and sample size

The study participants included 45 first-year medical (M1) students who volunteered for the Learning Community (LC) pilot program at the Medical College of Georgia. The LC pilot program was designed to study extracurricular methods to minimize burnout and foster passion in students throughout medical school. The program provides opportunities for up to 45 students of the 190 M1 student class to participate in guest lectures, patient encounters, reflective writing assignments, and group discussions for a couple of hours a week. Of the 45 M1 students in the LC program, 35 attended the 2-hour community forum and agreed to participate in the study (18 males and 17 females). This study was approved by Augusta University’s IRB. All study participants provided informed consent and participated voluntarily. The anonymity of data was maintained through de-identification, and data were stored over a secure server.

### Sampling procedure

A convenience sample of the 35 M1 student participants who attended the community forum was employed for this exploratory study (participation rate = 77.8%). Students were asked to reflect on their experience at the aforementioned 2-hour community forum in November 2018 by submitting an anonymously written reflection.

### Setting

The 2-hour open forum took place at a predominantly African-American/black local church in Augusta, Georgia. In addition to the 35 M1 student participants, over 50 community members attended and participated in the forum. Community members were asked to share their positive and negative experiences with physicians and the healthcare system and to describe the attributes they want to see in their physicians.

### Data collection

The qualitative reflection tool was developed by two researchers (NR-W and WP) and was based on previous qualitative studies on intersectionality.^8^^,^^10^ The tool consisted of the following five questions: (1) How did it feel meeting with people in the community? (2) What did you learn about what patients really want in a doctor? (3) Is how you are preparing to become a physician congruent with the expectations of the people you will serve? Why or Why not? (4) Based on what you heard, how are we fulfilling our social contract, i.e., are we meeting the healthcare needs of the local community? (5) What improvement to healthcare do you want to make through your leadership? Written reflections were submitted electronically to one researcher (WP) using the university-sanctioned storage cloud in December 2018 and subsequently de-identified.

### Data analysis

Two researchers (RV and SA) employed Glaser’s classic grounded theory, independently using substantive coding and initially analyzing with open coding to assess for emerging themes and followed with selective coding using the constant comparative method.^8^ Briefly, the reflections were coded by assigning keywords to textual data that were considered relevant to the aims of the study. The researchers then compared analyses and discussed differences. For differences that could not be agreed upon, a third researcher (NR-W) resolved discrepancies to confirm reliability. All 35 reflections were analyzed through this iterative process. The finalized coding themes were discussed thoroughly by all authors. To elevate the analysis, Taylor’s concept of the modern social imaginary was applied as an analytical lens to the themes that emerged from the data.^6^

## Results

We analyzed questions 2 – 4 from above for themes. The students identified that patients value physicians who are present, listen, physically touch them, and employ communication amongst the healthcare team. There were occasional differences between whether patients desire a physical medical touch (touch during the physical exam) (61% of responses) or a compassionate physical touch (6% of responses), with 33% not otherwise specifying. One student described compassionate touch as “small details like holding their hand, asking to adjust their pillows, or simply listening and showing true empathy” and emphasized the effect of this touch because it “[does] not take much time to do but [can] have a big impact on the patient-doctor relationship” In contrast, a medical physical touch, which students perceived to be more desirable in patients, is described as “[touch during] physical exams to see what might be wrong, instead of simply talking to the patient” (Participant 22). In other words, actually carrying out a hands-on physical exam.  Table 1 shows all seven themes identified from the student responses to question 2, what the students learned about what patients desire in a physician.

**Table 1:** Themes of responses to question 2 (N=35)

What did you learn about what patients want in a physician?	Number of Student Responses
Physician who Listens	22
Physical Touch	18
Present Physician	17
Communication among Healthcare Team	17
Compassion	16
Continuity of Care	6
Empathy	4

Question 3 (Table 2) asked the students which aspects of their medical school experience are helping them achieve the qualities that patients want in a physician. Of the 35 responses, 12 (33.3%) reported that the Physical Diagnosis course, where students learn skills for performing a physical exam and practice interviewing standardized patients, is helping them become a physician congruent with the expectations of the patients. Specifically, students felt “the importance of a physical exam and the valuable information that can be gained from it is always stressed during our Physical Diagnosis class.” (Participant 4) The second most common response was volunteering and community engagement (25%), which includes student-run health clinics and opportunities that students seek for themselves. Only 5.6% of students felt that the preclinical curriculum is helping prepare them for what patient’s desire, while 28.5% felt that emphasis on the preclinical curriculum was not aligned with what patients wanted in physicians. As one student noted,

“Right now, I do not believe I am preparing to become a physician [congruent with the expectations] of patients. All I am focused on is studying and passing my multiple-choice tests. Though I understand that I must grasp a basic foundation of medical knowledge, I do not think I am giving enough attention to the other aspects of my education, such as physical diagnosis and volunteering in clinics. It is in those areas that I get patient interaction and can truly practice listening to others, instead of only myself.” (Participant 7)

The final questions asked students to apply what they heard patients desire in their medical care to the care they have experienced or seen in the healthcare system. Ten students (28.6%) felt that physicians are fulfilling the social contract and meeting the needs of the local community; two of these students specified that physicians are fulfilling the social contract with the current resources available. One student noted, “for the most part, it seems that we are helping fulfill the needs of the community. However, we have some areas we need to work on like compassion, empathy, etc…” (Participant 22). However, the majority of students (94%) felt that the healthcare system needs to improve. Of these 33 students, 69.7% provided ideas of ways to change the system and allow physicians to fulfill better their social contract, based on what they heard the patients saying at the community meeting. Some examples listed were increasing access to care and addressing some of the social determinants of healthcare (Table 3). About one-third of the students (30.3%) felt that, as medical students, they were unable to make any changes to benefit the medical system as a whole. As one student explained,

“I believe that the majority of physicians are making a sincere effort to fulfill the social contract. However, I think that recent changes in medicine have made the doctor-patient interaction that is expected more difficult to attain. For example, the burden on doctors to document every detail of a patient visit electronically has shifted where the physician’s attention is placed. In addition, I feel that the shifting and expanding role of private insurance has altered the public’s perception of healthcare and physicians.” (Participant 29)

## Discussion

To our knowledge, this is the first study in the medical education literature that analyzed self-reflection data after interactions with patients in a community-embedded forum through the lens of modern social imaginary theory. Several studies have discussed curricular development, highlighting the complex interplay of patient and physician expectations,^11^^-^^13^ yet rarely include real patients to describe current healthcare problems facing their communities. In general, conversations with patients typically take place within health care environments.  Understanding the values and needs of the surrounding community can provide the impetus to adapt the patient-physician relationship, and additionally amend the “social contract” that has lost footing in recent years.

**Table 2:** Themes of responses to question 3 (N=35)

Is how you are preparing to become a physician congruent with the expectations of the people you will serve?	Number of Student Responses
Physical Diagnosis	12
Volunteering/Community Engagement	9
Communication Skills	4
Intersessions	3
Learning Community (LC)	3
Preclinical Curriculum	2
Problem Based Learning (PBL)	2
Cultural Competence	1

In this study, students emphasized how the local community members advocated for the humanistic side of medicine. Soft skills like active listening, compassion, and communication were highlighted while academic expertise and prestige were rarely mentioned. Additionally, students’ reflections underscored the importance of their clinical skills lab (Physical Diagnosis course) and co-curricular activities like volunteering and community engagement initiatives as a means to develop these important skill sets. Our results have significance for our own curriculum, to expand upon opportunities within the clinical skills lab to practice and assess humanistic medicine, and other medical schools to explore what curricular and co-curricular activities emphasize elements of patient-focused care.

**Table 3:** Themes of responses to question 5 (N=35)

Areas identified to improve health system	Number of Student Responses
Increasing quality of care provided	25
Increasing access to care	15
Improving social determinants of health	14
Systemic changes (e.g. Medicaid expansion, decreasing paperwork)	10
Regaining patient/community trust	10
Increasing physician education about community	8
Increasing patient education	4

Two salient implications result from this work. First, medical educators who champion humanistic medicine should partner with local community members to assess how their respective medical curriculum is meeting the perceived needs of the community. Although faculty who design and teach curricula that include developing the soft skills of medical students may be aware of the most innovative research and strategies, interfacing with the local community may provide the most important data regarding patient-physician relationships. The results of these efforts likely will influence medical education by emphasizing how physician training and community involvement can influence how the next generation of physicians practice medicine.

Second, our work demonstrates how the modern social imaginary theory can be incorporated as a lens to interpret the patient-physician relationship. Most recently, Loyola University Chicago Stritch School of Medicine developed an elective curriculum track for its students that explored the theory of social imaginary and emphasized the practices of Ignatian spirituality.^8^ Their work emphasized how medical student participants could reconceptualize the practice of medicine to change the students’ outlook and interactions with patients.^8^^,^^9^ Our study enhances this previous work by adding a direct dimension of real-patient interactions, feelings, and beliefs to the development of medical students’ social imaginaries.  In addition, our study begins to explore the students’ perception regarding how the formation of their professional identity aligns with the expectations of the society they will serve.

The limitations of this study include the size and selection of the church members and the LC students, as well as the limited time of the discussion. As an underserved area, the local church community of Mount Version Baptist is likely to have encountered more difficulties with accessing fair and adequate health care. Additionally, the community members in attendance the night of the forum may have been more willing to attend if they had strong personal connections, whether positive or negative, with the health care system. Another limitation included the LC participants; although our sample of medical students were randomly assigned participation in this community among their cohort of 190 members, the first year medical students have limited clinical experience. They may not have been able to ask the community members specific questions regarding their attitudes towards healthcare. The students may also have had variable personal experiences interacting with physicians in the community to our fourth question regarding healthcare workers’ social imaginary. On a similar note, the forum was scheduled for only two hours, which limited the opportunity for each community member to relay their experiences and wishes for interactions with physicians. The prospect of expanding the forum to more frequent and longer meetings would likely give medical students a more personalized narrative from the church members about the ideas and expectations they have of doctors.

Lessons learned from this forum have motivated faculty and students to plan future interactions between medical students and community members. One possibility is to expand the forum to reach more students and to invite physicians to attend. Creating a forum ensuring patient confidentiality would allow the community members to be honest and open without the fear of damaging their current physician

relationship. Continuing to host these meetings at a local church or community establishment would be the best way to increase community members level of comfort and promote freedom to share their experiences with the students. In addition to increasing the size and frequency of meetings, the medical school may consider implementing curricular changes reflecting the responses from this study. Curricular changes may include earlier involvement with patients and interacting with patients in the local community.  These changes will likely foster the growth and development of the students’ professional identity regarding patient-centered care before beginning their clinical responsibilities. Future research should explore how these interventions change the patient-physician relationship in local communities and how medical educators can upscale such interventions to accommodate more learners.

## Conclusions

Providing opportunities for medical students to explore the patient-physician relationship and humanist medicine as a means to develop their professional identity can be influenced positively by interactions with local community members. Identifying the importance of active listening and compassion as salient qualities of physicians is crucial for the development of future physicians. Both curricular and co-curricular experiences may provide fundamental opportunities for medical students to explore their personal social imaginary in medicine.

### Conflict of Interest

The authors declare that they have no conflict of interest.
